# Pomelo seed oil: Natural insecticide against cowpea aphid

**DOI:** 10.3389/fpls.2022.1048814

**Published:** 2022-11-08

**Authors:** Wei Ling, Kumaravel Kaliaperumal, Meiling Huang, Yan Liang, Zhigang Ouyang, Zhonggao Zhou, Yueming Jiang, Jun Zhang

**Affiliations:** ^1^ National Engineering Research Center of Navel Orange, Gannan Normal University, Ganzhou, China; ^2^ Unit of Biomaterials Division, Department of Orthodontics, Saveetha Dental College and Hospitals, Saveetha University, Chennai, India; ^3^ School of Chemistry and Chemical Engineering, Gannan Normal University, Ganzhou, China; ^4^ South China Botanical Garden, Chinese Academy of Science, Guangzhou, China

**Keywords:** cowpea aphids, pomelo seed oil, chemical composition, insecticidal, natural compounds

## Abstract

Cowpea aphid (*Aphis craccivora* Koch) is a plant pest that causes serious damage to vegetable crops. Extensive use of synthetic chemical pesticides causes deleterious effects on consumers as well as the environment. Hence, the search for environmentally friendly insecticides in the management of cowpea aphids is required. The present work aims to investigate the aphicidal activity of pomelo seed oil (PSO) on cowpea aphids, the possible insecticidal mechanisms, its chemical constituent profile, as well as the toxicity of its primary compounds. The results of the toxicity assay showed that PSO had significant insecticidal activity against aphids with a 72-hour LC_50_ value of 0.09 μg/aphid and 3.96 mg/mL in the contact and residual toxicity assay, respectively. The enzymatic activity of both glutathione S-transferase (GST) and acetyl cholinesterase (AChE) significantly decreased, as well as the total protein content, after PSO treatment, which suggested that the reduction of AChE, GST, and the total protein content in aphids treated with PSO might be responsible for the mortality of *A. craccivora*. The GC-MS analysis revealed that PSO contained limonene (22.86%), (9Z,12*Z*)-9,12-octadecadienoic acid (20.21%), *n*-hexadecanoic acid (15.79%), (2*E*,4*E*)-2,4-decadienal (12.40%), and (2*E*,4*Z*)-2,4-decadienal (7.77%) as its five major compounds. Furthermore, (9Z,12*Z*)-9,12-octadecadienoic acid showed higher toxicity to aphids than both PSO and thiamethoxam (positive control). This study emphasized the potential of PSO as a natural plant-derived insecticide in controlling cowpea aphids and also provided a novel approach for the value-added utilization of pomelo seed.

## Introduction

Pest causes extensive damage to the crops like grains and vegetables by directly consuming the plant parts like shoots, seeds, flowers, and plant sap (Wielkopolan et al., 2021), and creates huge economic losses to the farmers in terms of loss of yield and low-quality food products which cannot be commercialized in the market (Mburu et al., 2020). In addition, its waste excretion is deleterious to plants and humans who consume the plants. It has been reported that every year almost 5-30% of total global agricultural yield is affected by pest infestation (Prusky, 2011). *Aphis craccivora* Koch (Homoptera: Aphididae) commonly known as cowpea aphid, is distributed worldwide, but particularly rampant in warm temperate and tropical regions (Dolma et al., 2021). Cowpea aphid is a polyphagous pest since it feeds on over 80 plant families with a preference for the family Fabaceae. It sucks plant juice with sharp mouthparts, resulting in the deterioration of plant nutrition and even defoliation (Arcaya et al., 2017). Apart from that, it produces a large amount of honeydew, which encourages the formation of sooty mold and slows the growth of the plant (Paul and Das, 2021). Additionally, Aphid acts as a vector of about 30 plant viruses including the Tobacco Etch Virus (TEV) and the Papaya Ring Spot Virus (PRSV), both of which exclusively damage the vegetative and edible crops (Wang et al., 2006).

The most successful method of preventing cowpea aphid infestations is the administration of chemical synthetic pesticides. However, the continued use of this type of pesticide harms the ecosystem and promotes the emergence of mutants that are resistant to it (Slater et al., 2012; Richardson et al., 2022). In addition, the majority of synthetic pesticides are not biodegradable, so when rains wash them away, they could contaminate both freshwater bodies and groundwater (Gavrilescu, 2005; Baran et al., 2022). Thus, there is a need to search for alternatives that have limited adverse effects on the environment and are effective against target pests. One such option is the use of botanical pesticides, which have exhibited some control of crop pests (Opolot et al., 2006; Tembo et al., 2018; Ngegba et al., 2022). Essential oils from aromatic plants and their major constituents, were considered to be alternatives to conventional synthetic pesticides for controlling many pests, due to several advantages including rapid degradation, low residual, and versatile mechanism of action (Czerniewicz et al., 2018; Mostafiz et al., 2020; Zhou et al., 2021). For example, it was reported that the essential oil of *Hemizygia petiolata* Ashby containing high levels (>70%) of the sesquiterpene (*E*)-β-farnesene, significantly reduced the numbers of pea aphid (*Acyrthosiphon pisum*) in the field-plot experiment (Bruce et al., 2005). In addition to essential oils, many other secondary metabolites such as alkaloids, triterpenoids, steroids and flavonoids present in plants were found showing insecticidal effects as well (Rattan, 2010; Boulogne et al., 2012; Vite-Vallejo et al., 2018). Worthy of note, the crude alkaloid obtained from *Sophora flavescens* was registered as a commercial botanical pesticide in China, which not only can efficiently control aphids, caterpillars and other pests on the tomato plant, but also can improve the growth and development of this plant (Xiong et al., 2016).

The essential oil obtained from pomelo peel is generally recognized as safe and is rich in aromatic substances that can be used as flavor and fragrance material in the food flavoring, beverage, pharmaceutical, and cosmetic industries (Liu et al., 2017). In addition, the essential oil from pomelo peel has been reported to possess a broad spectrum of bioactivities, such as anti-inflammatory, antioxidant, and antimicrobial effects (Uysal et al., 2011; Xiao et al., 2021), and contain monoterpenoid involving limonene, β-myrcene and β-pinene as its major chemical compositions (Uysal et al., 2011; He et al., 2019). However, the chemical composition and biological activity of pomelo seed oil (PSO) are largely unknown. Most recently, Pu et al. (2022) reported that the *Majia* pomelo seed oil had 39.32% lipid mainly composed of linoleic acid and oleic acid, and was not toxic to normal liver LO2 cells. In one of our previous studies, the ethyl acetate extract from pomelo seed was found to exhibit good antioxidant and herbicidal activities and contain naringin, deacetylnomilin, limonin, nomilin, and obacunone as its main components (Ling et al., 2021).

The present study is aimed to evaluate the insecticidal activity of pomelo seed oil (PSO) against cowpea aphids and its possible mechanism of action. Meanwhile, the chemical composition of PSO is investigated by GC-MS analysis and the primary compounds in PSO were then evaluated for aphicidal effect. Since pomelo seed is generally considered a waste product of the agriculture industry, the present study provided a novel approach to the value-added utilization of pomelo seed.

## Materials and methods

### Chemical reagents

All chemicals used are of analytical grade. Bovine serum albumin, 1-chloro-2,4-dinitrobenzene (CDNB), disodium phosphate, Coomassie brilliant blue (G-250), acetylthiocholine iodide (ATChI), and L-glutathione reduced (GSH) were obtained from Macklin Biochemical Co. Ltd (Shanghai, China). 5,5′-dithiobis-(2-nitrobenzoic acid) (DTNB), phosphate buffer saline (PBS) buffer, and C_7_-C_40_ *n*-alkane mixture were acquired from Sigma-Aldrich China Co. Ltd (Shanghai, China). Limonene, (9Z,12*Z*)-9,12-octadecadienoic acid, *n*-hexadecanoic acid, and (2*E*,4*E*)-2,4-decadienal were purchased from Solarbio Science and Technology Co., Ltd. (Beijing, China).

### The cultivation of cowpea aphids

Cowpea aphids (*A. craccivora*) adults were picked from the farm yard nearby Gannan Normal University, China. The aphids were collected from the infested and pesticides-unsprayed cowpea plants. The aphids were reared on fresh green cowpea plants and maintained in a greenhouse at 25 ± 2°C with 70 ± 5% relative humidity and 14:10 h (L:D) photoperiod by using the method reported previously (Yeo et al., 2003). Uniform-aged nymphs of aphids (4d old) were selected for all subsequent experiments.

### The preparation of pomelo seed oil

Commercially mature Shatian pomelos were harvested from an orchard in Nankang, Jiangxi province, China. The fruits were identified as Shatian pomelos (*Citrus grandis* Osbeck cv. shatian) by Prof. Balian Zhong. The pomelo seeds were obtained from the fresh fruits by hand and then dried at 50°C in an oven until maintained constant weight. A voucher specimen of this dry PS (NPR-201808) was deposited in the School of Life Science, Gannan Normal University, China. Before extraction, the dry seeds were cut into small pieces by scissors. The PSO was prepared by heat extraction according to a previous method with some modifications (Beveridge et al., 2005). Briefly, dried and chopped pomelo seeds (2 kg), were extracted two times by refluxing with 95% ethanol at 80°C, 2 h for each time, and then concentrated at reduced pressure to obtain crude extract (250 g). 1.0 L of distilled water was added to suspend the crude extract and then extracted two times with petroleum ether (1.0 L x 2). The combined petroleum ether extract is concentrated by vacuum concentration at 30°C to give PSO (68.08 g) with a yield of 3.4% based on the weight of the dry seed. PSO is immediately stored at 4°C in the refrigerator until use.

### Residual toxicity assay

Residual toxicity was assessed using the methodology reported previously with some modifications (Dolma et al., 2021). The sample was dissolved in a 1% Tween-80 aqueous solution to prepare a stock solution (80 mg/mL), which was then subjected to two-fold dilution with distilled water to give different concentrations (40, 20, 10, 5, and 2.5 mg/mL) of test solutions. Freshly harvested green cowpea pods (*Vigna unguiculata* L.) were washed with distilled water and cut into 4 cm-long pieces, which were then immersed in the test solutions for 5 s. After that, the treated cowpea pods were taken out, dried naturally at room temperature for 1 h, and then placed in a Petri dish (12.5 cm in diameter) lined with moistened filter paper. The cowpea aphids starved for 12 h were then put into the Petri dish. Each Petri dish contained 30 aphids and four pieces of cowpea pods pretreated with samples. The Petri dishes were placed in a light incubator with a temperature of 25 ± 2°C, relative humidity of 70 ± 5%, and a light time of L: D = 14:10 h. The death of cowpea aphid was recorded at 12, 24, 36, and 72 h after the treatment. The cowpea aphid body was smoothly touched with a soft brush under the dissecting microscope, and those who responded to touching were considered to be alive. All the assays were performed in triplicate, and the means ± SD (standard deviation) were calculated. The 1% Tween-80 aqueous solution was used as a blank control. The mortality was calculated as follows:


Mortality(%)=(number of dead aphids/number of all aphids tested)×100

All mortality data were corrected by Abbott’s formula and subjected to log-probit analysis to calculate LC_50_ values which were expressed as the concentration (mg/mL) causing 50% mortality of aphids.

### Contact toxicity assay

The contact toxicity of samples on cowpea aphids was assessed by following the methodology reported previously (Zhou et al., 2016), with slight modifications. The sample was dissolved in a 1% Tween-80 aqueous solution to prepare a stock solution (80 mg/mL), which was then subjected to two-fold dilution with distilled water to give different concentrations (40, 20, 10, 5, and 2.5 mg/mL) of test samples. Each test solution (0.05 μL) was then topically applied on the pronotum of cowpea aphids with a topical applicator (Model- PAX100-2, Burkard Scientific, UK). 30 cowpea aphids were used for each concentration tested. After the treatment, a soft brush was used to transfer the cowpea aphids to a Petri dish (12.5 cm in diameter) lined with moistened filter paper and containing fresh cowpea pods. The Petri dish was then placed in a light incubator with a temperature of 25 ± 2°C, relative humidity of 70 ± 5%, and a light time of L: D = 14:10 h. The death of cowpea aphid was recorded at 12, 24, 36, and 72 h after the treatment. The cowpea aphid was smoothly touched with a soft brush under the dissecting microscope, and those who responded to touching were considered to be alive. All the assays were performed in triplicate, and the means ± SD (standard deviation) were calculated. The 1% Tween-80 aqueous solution and thiamethoxam were used as a blank and positive control, respectively. The corrected mortality and LC_50_ were calculated by the same method as that on the residual toxicity assay. The LC_50_ values were expressed as the amount per aphid received (μg/aphid) which caused 50% mortality of aphids (Ma et al., 2018).

### Preparation of tissue samples of aphids

The preparation of tissue samples of aphids for biochemical assays was done following the methodology reported previously with minor modifications (Czerniewicz et al., 2018). In brief, 50 cowpea aphids treated with PSO at the amounts of 0.56 μg/aphid (the 24-hour LC_30_ in contact toxicity assay) or 1.28 μg/aphid (the 24-hour LC_50_ in the contact toxicity assay), were transfer to a Petri dish (12.5 cm in diameter) lined with moistened filter paper and containing fresh cowpea pods. The petri dish was then placed in a light incubator with a temperature of 25 ± 2°C, relative humidity of 70 ± 5%, and a light time of L: D = 14:10 h. After 4, 8, 12, and 24 h of treatment, 10.0 mg of aphids were then taken out and homogenized in 1 mL of PBS buffer (0.04 M, pH = 7.4) at 0°C. The resultant homogenate was subjected to centrifugation at 1500×g for 10 min at 4°C, and the supernatant was collected and used to determine the total protein contents, GST, and AChE activities. All the experiments were conducted in triplicate and the aphids treated with 1% Tween-80 aqueous solution were used as blank control.

### Determination of total protein contents

The total protein contents of cowpea aphids after PSO exposure were measured according to the previous method (Elbanhawy et al., 2019). The reaction solution contained 10 μL of the supernatant obtained above and 270 μL of Coomassie brilliant blue (G-250). After the mixture was incubated for 5 min at 25°C, the OD (optical density) was immediately recorded by using a Tecan Spark^®^ 10M microplate reader (Männedorf, Switzerland) at 595 nm. The total protein content was calculated based on an albumin standard curve and expressed as mg per gram of aphid (mg/g). All the assays were performed in triplicate, and the means ± SD (standard deviation) were calculated.

### Determination of GST activity

The glutathione S-transferase (GST) activity was tested following the method reported previously with some modifications (Vontas et al., 2001). To a well of 96-well plate, the enzyme solution (10 μL) was added, followed by the addition of GSH (100 μL, 6 mM in PBS) and CDNB (100 μL, 1.2 mM in 1% ethanol/PBS (v/v)) in sequence. The OD value at 340 nm was immediately recorded at regular intervals of 30 s for 40 min by using a Tecan Spark^®^ 10M microplate reader (Männedorf, Switzerland). For the blank control, 10 μL of PBS buffer (0.04 M, pH = 7.4) was used instead of enzyme solution. The relative enzymatic activity of GST was expressed as the change of OD value per 20 min (ΔOD/20 min) in the linear part of the curve of time versus OD value. All the experiments were conducted in triplicate.

### Determination of AChE activity

The acetyl cholinesterase (AChE) activity was analyzed following the methodology reported previously with slight modifications (Bezerra da Silva et al., 2016). In brief, To a well of 96-well plate, the enzyme solution (50 μL) was added, followed by the addition of ATChI (150 μL, 20 mM in PBS) and DTNB (50 μL, 1.0 mM in PBS) in sequence. The OD value at 415 nm was immediately recorded by using a Tecan Spark^®^ 10M microplate reader (Männedorf, Switzerland), at regular intervals of 30 s for 40 min at 30°C. For the blank control, 50 μL of PBS buffer (0.04 M, pH = 7.4) was used instead of enzyme solution. The relative enzymatic activity of AChE was expressed as the change of OD value per 20 min (ΔOD/20 min) in the linear part of the curve of time versus OD value. All the experiments were conducted in triplicate.

### Chemical composition analysis of PSO by GC-MS

The chemical composition of PSO was analysed by gas chromatography-mass spectrometry (GC-MS) using a previous method with minor modifications (Zhang et al., 2022). The Agilent 7890B gas chromatography coupling with an Agilent mass spectrometer detector (Agilent Technologies, Santa Clara, CA, USA) was used. The non-polar column HP-5 (30.00 m × 0.25 mm × 0.25 µm) was employed with high-purity helium as the carrier gas. The inlet temperature was 280°C and the carrier gas flow rate was 1 mL/min. The column oven temperature was programmed to increase at the rate of 4°C/min from 80 to 280°C, where the temperature was held for an additional 7 min. The ionization energy was 70 eV and the acquisition mass range was 40-550 *m/z*. A mixture of C_7_–C_40_ *n*-alkane was added to the PSO and analyzed under the same conditions mentioned above to calculate the retention indices (RI) of the constituents. The chemical components of PSO were identified by the comparison of their RIs with literature values reported at the website (http://www.flavornet.org/flavornet.html) and mass spectra with the National Institute of Standards and Technology (NIST) database. The four primary volatiles were further determined by comparing with the commercially available standard compounds. The relative amount of individual components in PSO was calculated in peak areas using the normalization method without correction factors, and expressed as a percentage of total peak areas.

### Statistical analysis

Data were expressed as means ± standard deviation (SD) of three replicates. One-way analysis of variance (ANOVA) and Tukey’s test were used for data analysis at a significance level of p < 0.05 by SPSS 23 (IBM Corporation, Armonk, NY, USA). SigmaPlot software (version 14.0, San Jose, CA, USA) was employed to create figures.

## Results

### Residual and contact toxic effects of PSO

The present study revealed that PSO had excellent toxicity on cowpea aphids ingesting the PSO-treated diet. As shown in **Table 1**, the LC_50_ values were 51.53, 38.19, 4.36, and 3.96 mg/mL for 12, 24, 36, and 72 h of exposure time in the residual toxicity assay, respectively. In the meantime, the toxic effect of PSO on cowpea aphids assessed by contact toxicity method revealed that the LC_50_ values were 2.73, 1.28, 0.40, and 0.09 μg/aphid at the treatment period of 12, 24, 36, and 72 h, respectively (**Table 2**). The mortality was high when the treatment period was prolonged and the dose of PSO increased. These results suggested that the aphids could be exposed to toxicity by means of contacting PSO or ingesting PSO-contaminated food materials.

**Table 1:** The residual toxicity of PSO (pomelo seed oil) against *Aphis craccivora*.

Time (h)	LC_50_ (95% CI) (mg/mL)	Slope ± S.E.	χ^2^ (*d.f.*)	*P*-value
12	51.53 (37.21-63.83)	1.82 ± 0.22	5.54 (13)	0.96
24	38.19 (25.14-48.86)	1.18 ± 0.17	10.27 (13)	0.67
36	4.36 (2.38-6.56)	1.19 ± 0.17	12.65 (13)	0.47
72	3.96 (2.49-5.24)	1.57 ± 0.20	9.35 (13)	0.36

LC_50_ value was determined by log-probit analysis; 95% CI, confidence interval at 95%; S.E., standard errors; χ^2^, chi square; d.f., degrees of freedom.

**Table 2:** The contact toxicity of PSO (pomelo seed oil) against *Aphis craccivora*.

Time (h)	LC_50_ (95% CI) (μg/aphid)	Slope ± S.E.	χ^2^ (*d.f.*)	*P*-value
12	2.73 (1.87-4.800)	1.65 ± 0.20	14.54 (13)	0.33
24	1.28 (0.92-1.99)	1.55 ± 0.17	19.59 (13)	0.11
36	0.40 (0.21-0.59)	1.63 ± 0.18	3.67 (13)	0.99
72	0.09 (0.01-0.22)	1.37 ± 0.23	6.83 (13)	0.91

LC_50_ value was determined by log-probit analysis; 95% CI, confidence interval at 95%; S.E., standard errors; χ^2^, chi square; d.f., degrees of freedom.

### Effect of PSO on AChE, GST activities, and total protein contents of aphids

**Figure 1:**
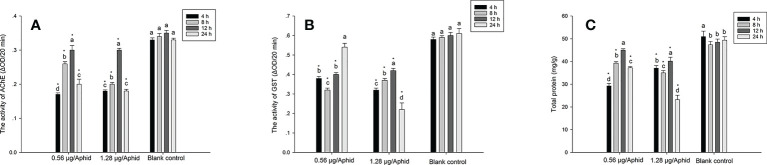
Effect of PSO (pomelo seed oil) at the dose of 0.56 μg/aphid (LC_30_) or 1.28 μg/aphid (LC_50_) on some biochemical properties of *Aphis craccivora*, **(A)**: *In vivo* AChE activity; **(B)**: *In vivo* GST activity; **(C)**: Total protein content. The different lowercase letters within the same treatment group indicate significant difference (p < 0.05). The * indicates significant difference (p < 0.05) compared to the corresponding blank control group at the same time interval.

The current finding suggested that PSO treatment considerably decreased aphid AChE activity. At 4, 8, 12, and 24 hours after exposure, **Figure 1A** demonstrated that the control group had considerably higher AChE activity than the group that had received 0.56 or 1.28 μg/aphid of PSO. Meanwhile, the AChE activity of aphids exposed to PSO steadily increased during the first 12 hours, and then it reduced subsequently, according to the current study.

GST enzyme is involved in the detoxification process of insects when they are exposed to toxic exogenous compounds, and it is also highly related to the development of insecticide resistance in insects (Li et al., 2013). The present result indicated that PSO significantly decreased the GST activity in both doses (0.56 and 1.28 μg/aphid) treatment groups when compared to the control group (**Figure 1B**) (p < 0.05). In the low dose (0.56 μg/aphid) treatment group, the variation of GST activity displayed a trend toward a decrease at the initial stage (first 8 h), followed by an increase during the late period. However, in the high dose (1.28 μg/aphid) treatment group, the GST activity gradually increased during the first 12 h of exposure time, and then significantly decreased afterward.

Protein synthesis is an important biological function in all cells and living organisms since it is the building block of tissues (Avila et al., 2011). The present result indicated that the total protein content in aphids treated with PSO significantly decreased in comparison with the control group (**Figure 1C**). The protein content of aphids in the control group ranged around 47.36-50.94 mg/g, which was significantly (p < 0.05) higher than that of the PSO-treated groups, being 29.25-45.01 (0.56 μg/aphid treatment), and 23.20-40.15 mg/g (1.28 μg/aphid treatment), respectively. The lowest protein content was recorded as 23.20 mg/g at the dose of 1.28 μg/aphid after 24 h of treatment, whereas the corresponding control group was 49.35 mg/g.

### Gas chromatography and mass spectrometry analysis of PSO

Limonene (22.86%), (9*Z*,12*Z*)-9,12-octadecadienoic acid (20.21%), *n*-hexadecanoic acid (15.79%), (2*E*,4*E*)-2,4-decadienal (12.40%), and (2*E*,4*Z*)-2,4-decadienal (7.77%) are the five most prevalent compositions among the 18 primary compounds found in PSO by GC-MS analysis (**Figure S1 in Supplementary material**). Additionally, the GC-MS data showed that long-chain fatty acid (ester) (40.58%) was the main category of ingredients in PSO, followed by monoterpene (27.71%), aldehyde (21.35%), long-chain alkene (4.11%), and ketone (1.89%). (**Table 3**).

**Table 3:** Chemical compositions of PSO (pomelo seed oil) by GC-MS.

No	RT^a^ (min)	Compositions	Percentage (%)(%)	RI^b^	RI^c^	RI^d^
1	5.47	Limonene	22.86 ± 0.32	1028	1030	1031
2	12.961	(*E*)-2-Decenal	1.18 ± 0.07	1262	1260	
3	14.039	(2*E*,4*Z*)-2,4-Decadienal	7.77 ± 0.11	1294	1291	
4	14.814	(2*E*,4*E*)-2,4-Decadienal	12.40 ± 0.15	1317	1317	1320
5	17.151	Tetradecene	0.64 ± 0.02	1389	1381	
6	18.061	Caryophyllene	1.47 ± 0.03	1417	1417	
7	20.369	Eremophilene	3.38 ± 0.12	1491	1486	
8	23.242	Cetene	0.65 ± 0.03	1588	1587	
9	25.416	(6*Z*,9*E*)-Heptadecadiene	1.28 ± 0.09	1665	1667	
10	25.626	8-Heptadecene	0.83 ± 0.04	1672	1670	
11	28.727	1-Octadecene	0.71 ± 0.05	1787	1788	
12	29.193	Nootkatone	1.18 ± 0.05	1805	1805	
13	30.091	6,10,14-Trimethyl-2-pentadecanone	0.71 ± 0.02	1835	1835	
14	32.125	*n*-Hexadecanoic acid methyl ester	1.11 ± 0.06	1922	1920	
15	33.326	*n*-Hexadecanoic acid	15.79 ± 0.29	1971	1971	1972
16	33.763	*n*-Hexadecanoic acid ethyl ester	2.42 ± 0.12	1989	1993	
17	36.124	(9*Z*,12*Z*)-9,12-Octadecadienoic acid methyl ester	1.05 ± 0.03	2091	2092	
18	37.26	(9Z,12*Z*)-9,12-Octadecadienoic acid	20.21 ± 0.39	2142	2144	2142
		Monoterpene	27.71 ± 0.42			
		Aldehyde	21.35 ± 0.46			
		Long chain alkene	4.11 ± 0.12			
		Ketone	1.89 ± 0.05			
		Long chain fatty acid (ester)	40.58 ± 0.58			
		Total	95.64 ± 0.69			

^a^, Retention time; ^b^, Retention indices (RI) observed in experiment; ^c^, RI reference data obtained from the website http://www.flavornet.org/flavornet.html; ^d^, Retention indices (RI) observed from the standard compounds.

### Contact toxicity of four primary compounds in PSO

Four primary and commercially available PSO compounds, including limonene, (9*Z*,12*Z*)-9,12-octadecadienoic acid, *n*-hexadecanoic acid, and (2*E*,4*E*)-2,4-decadienal, were tested for aphicidal activity using contact toxicity assay to further identify the compounds underlying the insecticidal effect of PSO. As shown in **Table 4**, among all the compounds tested, (9Z,12*Z*)-9,12-octadecadienoic acid exhibited the best aphicidal activity with an LC_50_ value of 0.58 μg/aphid after 24 h of exposure time, which was more potent than the positive control thiamethoxam (LC_50_ = 1.55 μg/aphid) and PSO (LC_50_ = 1.28 μg/aphid). The compound (2*E*,4*E*)-2,4-decadienal also demonstrated potent toxicity toward aphids with an LC_50_ value of 5.28 μg/aphid, while slightly lower potential in comparison with PSO. However, neither limonene (LC_50_ = 150.78 μg/aphid) nor *n*-hexadecanoic acid (LC_50_ = 22.91 μg/aphid) displayed considerable contact toxicity toward aphids.

**Table 4:** The contact toxicity of primary compounds in PSO (pomelo seed oil) against *Aphis. craccivora* Koch after 24h treatment.

Compounds	LC_50_ (95% CI) (μg/aphid)	Slope ± S.E.	χ^2^ (*d.f.*)	*P*-value
Limonene	150.78 (82.35-750.21)	0.45 ± 0.08	1.97 (13)	0.99
(2*E*,4*E*)-2,4-Decadienal	5.28 (2.36-8.64)	0.78 ± 0.07	4.92 (13)	0.97
*n*-Hexadecanoic acid	22.91 (14.20-46.84)	0.97 ± 0.10	3.14 (13)	0.99
(9Z,12*Z*)-9,12-Octadecadienoic acid	0.58 (0.49-0.78)	1.09 ± 0.07	4.04 (13)	0.98
Thiamethoxam (positive control)	1.55 (1.24-1.99)	1.22 ± 0.08	5.86 (13)	0.95

LC_50_ value was determined by log-probit analysis; 95% CI, confidence interval at 95%; S.E., standard errors; χ^2^, chi square; d.f., degrees of freedom.

## Discussion

An eco-friendly and biocontrol approach for pest management by using plant secondary metabolites as insecticides (repellents, anti-feedants, and toxicants) has been increasingly popular in recent years (Tlak and Dar, 2021). In the present study, pomelo seed oil (PSO) was assessed for insecticidal activity against the pest cowpea aphid (*A. craccivora*) by using both residual and contact toxicities assays. Meanwhile, the influence of PSO on AChE, GST activity, and the total protein contents of aphids was also evaluated. Furthermore, the chemical compositions of PSO were analyzed by GC-MS studies and the four primary compounds present in PSO were tested for aphicidal activity as well. Laboratory studies on essential oils (EOs) and their components for use in eradicating pests have risen recently. According to a review of the literature, several recent studies have examined how EOs affect aphids and pests that live on stored products (Abdelaal et al., 2021; Al-Harbi et al., 2021; Sayed et al., 2022), but few research has specifically examined the aphicidal efficacy of citrus EOs. Most recently, Alotaibi et al. (2022) discovered that *Citrus aurantium* L. EO had greater toxicity against pomegranate and grapevine aphids, with LC_50_ of 0.37 and 0.82 μL/mL, respectively, after 48 h of application.

The present result revealed that PSO exhibited excellent residual toxicity to *A. craccivora* with a 72-hour LC_50_ of 3.96 mg/mL, which was in agreement with the findings of several previous studies. Dolma et al. (2021) reported that the 96-hour LC_50_ of crude extract of *Trillium govanianum* was 3709.1 mg/L against *A. craccivora*. The *n*-hexane fraction of *Eupatorium adenophorum* and *Ageratum houstonianum* showed good residual toxicity to *A. craccivora* with an LC_50_ being 2881 and 2590 mg/L, respectively, after 96 h of treatment (Adebisi et al., 2019). In another similar study, the saponins clematoside S (72-hour LC_50_ = 2.3 mg/mL) and clematograveolenoside A (72-hour LC_50_ = 3.2 mg/mL), isolated from *Clematis graveolens* were found highly effective against *A. craccivora* (Rattan et al., 2015). In our present research, the contact toxicity assay also revealed that PSO exhibited significant toxicity to aphids, with an LC_50_ of 0.09 μg/aphid after 72 h of treatment. Similarly, in a previous study, the total crude alkaloids in *Sophoraalo pecuroides* L. exhibited excellent contact toxicity to aphids with an LC_50_ of 0.02 μg/aphid against *A. craccivora*, 0.093 μg/aphid against *A. citricola*, and 0.117 μg/aphid against *A.* sp, after 24h of treatment (Ma et al., 2018). Consistent with the present study, essential oil from the fresh leaf of *Citrus hystrix* proved toxic to *A. gossypii* Glover, resulting in 50.4% mortality at the concentration of 3 μL/L air after 24 h of fumigation (Pumnuan et al., 2017).

The mode of action for most insecticides can be classified into three categories, namely, neurotoxins, growth regulators, and respiratory inhibitors (Song and Scharf, 2008). To determine the possible insecticidal mechanism of PSO, the influence of PSO on both AChE and GST enzymes was evaluated in the present study. The result indicated that PSO caused a pronounced reduction in the activity of these two enzymes. Similarly, the activities of these two enzymes (AChE and GST) in the aphid *Myzus persicae* were significantly inhibited at the initial stage, and then induced to restore to some extent with prolonged processing time after being treated with extracts from *Illicium verum* fruit (Zhou et al., 2016). AChE is essential for normal signal transduction, it influences the neural impulses by catalytic hydrolysis of acetylcholine (Bezerra da Silva et al., 2016). In the present study, PSO caused a significant reduction in AChE activity at the dose of both 0.56 μg/aphid (LC_30_) and 1.28 μg/aphid (LC_50_) during the whole treatment period. Therefore, PSO probably had neurotoxic effects on aphids (*A. craccivora*). In line with the present results, many botanical aphicides were reported to exert their insecticidal effects by targeting AChE enzyme (Zhou et al., 2016; Li et al., 2022). In particularly, lots of aphicidal EOs or their primary components were AChE inhibitors, for example, the EOs derived from Asteraceae family plants exhibited insecticidal activity to aphid *Myzus persicae* by inhibiting the activity of the AChE enzyme (Czerniewicz et al., 2018). In addition, methyl benzoate (MB), a volatile compound reported in several plants extracts, was determined to be toxic to cotton aphids (*A. gossypii* Glover) by decreasing the AChE activity up to 65% (Mostafiz et al., 2020).

GST exerts a pivotal part in the detoxification of insecticides in insects, which can combine with insecticidal molecules *via* chelation, or remove the lipid metabolites induced by insecticidal material, thus protecting tissues from oxidative damage (Zhang et al., 2013). Similar to the effect on AChE, PSO treatment significantly decreased GST activity. The present results suggested that the reduction of GST and AChE activities in *A. craccivora* may be responsible for the aphids mortality caused by PSO treatment, supportive of the potent application of PSO as a natural aphicide. In agreement with the present study, the activities of both AChE and GST in the aphid *Myzus persicae* and *Sitophilus zeamais* were notably decreased by the treatment of fruit extracts of *Illicium verum*, as compared with the control (Li et al., 2013; Zhou et al., 2016). Elbanhawy et al. (2019) reported that the methanol extract of *Purpureocillium lilacinum* greatly reduced the GST enzyme activity in *A. gossypii*, thus influencing its growth and metabolism. Contrary to the present study, the GST activity in the aphid *Myzus persicae* increased after the treatment with essential oils from *Santolinachamae cyparissus* and *Achillea millefolium* (Czerniewicz et al., 2018). Similarly, GST activity and the total proteins in the grain aphid S*itobio navenae* significantly increased with the treatment of catechol and gramine, both of which were deleterious to aphids (Zhang et al., 2013). This apparent discrepancy might be due to the different dose treated and the exposure time. It was reported that the GST activity of *Varroa destructor* changed depending on the dosage of the oil treated. The GST activity of *Varroa destructor* increased significantly at a low dosage (0.1 μL) when exposed to clove oil, but decreased at a higher dosage (1.0 μL) (Li et al., 2017). Worthy of note, in addition to GST, two other major detoxification enzymes involving cytochrome P450 oxidase (P450) and carboxylesterase (CarE), play critical roles in insecticide metabolism as well (Sharma et al., 2018). In this study, only the effect on GST was investigated. Therefore, further study is required to determine whether the PSO affects the enzymatic activities of both P450 and CarE, to achieve a more complete understanding of the response of *A. gossypii* to PSO.

Growth and metamorphism in insects are highly influenced by the ecdysone hormone as well as protein level in tissues (Avila et al., 2011). The total protein content of *A. craccivora* highly decreased after being treated with PSO according to our current study. This reduction might be attributable to the insect adaptation to reduce the stress obtained or response to the treatment of PSO by down-regulating the expression of some protein-related genes. Similar results were obtained in aphid *A. gossypii* treated with methanolic extracts of *Cladosporium cladosporioides* and *Purpureocillium lilacinum* (Elbanhawy et al., 2019). In the present study, PSO caused a significant reduction in the activity of AChE and GST. This reduction might be related to the decrease of the total protein content in *A. craccivora*. Similarly, Elbanhawy et al. (2019) reported that the remarkable reduction in the activity of α- and β-esterase enzymes was associated with the decrease of enzyme amount in *A. gossypii* treated with the extracts of *Purpureocillium lilacinum*. Therefore, we assumed that the PSO might down-regulate the expression of some genes related to both AChE and GST proteins, or inhibit the catalytic ability of these two enzymes, thus resulting in the reduction of enzymatic activity of these two enzymes.

As per the literature review, there are very few studies investigating the chemical composition of pomelo seed oil. Wan and Xiao (2008) reported that the pomelo seed oil contained more than 90% of fatty acids mainly involving linolenic acid, linoleic acid, oleic acid and palmitic acid. However, the chemical compositions of essential oil from pomelo peel were comprehensively investigated previously. For instance, limonene (55.92%), β-myrcene (31.17%), and β-pinene (3.16%) were reported as the primary components of essential oil from pomelo cv. Guan Xi (He et al., 2019). Meanwhile, limonene was observed as the dominant (88.6%) component, followed by β-Pinene (1.2%), linalool (0.7%), and α-terpinene (1.0%) in the essential oil obtained by hydrodistillation from the peel of grapefruit (*Citrus Paradisi.* L) (Uysal et al., 2011). In the present study, 18 major compounds were identified in PSO for the first time, including limonene (22.86%), (9Z,12*Z*)-9,12-octadecadienoic acid (20.21%), *n*-hexadecanoic acid (15.79%), (2*E*,4*E*)-2,4-decadienal (12.40%), and (2*E*,4*Z*)-2,4-decadienal (7.77%) as the five most abundant compounds.

Limonene is an aromatic compound present in all citrus fruit which is widely used as a fragrant agent in many cosmetics and also displays herbicidal and insecticidal effects (Karr and Coats, 1988; Kim et al., 2013). However, the present results revealed that limonene (LC_50_ = 150.78 μg/aphid) was not a potent aphicide. (9Z,12*Z*)-9,12-octadecadienoic acid and *n*-hexadecanoic acid are long-chain fatty acids, both of which were highly toxic to whiteflies and mites (Wagan et al., 2018). Furthermore, *n*-hexadecanoic acid is toxic to the fourth larval stage of *Aedes aegypti*, *Culex quinquefasciatus*, and *Anopheles stephensi*, with LC_50_ values of 57.23, 129.24, and 79.58 ppm, respectively (Rahuman et al., 2000). Our results are consistent with these previous reports. In the present research, (9Z,12*Z*)-9,12-octadecadienoic acid (LC_50_ = 0.58 μg/aphid) containing two unsaturated double bonds exhibited significantly higher aphicidal activity than *n*-hexadecanoic acid (LC_50_ = 22.91 μg/aphid), suggesting that the presence of unsaturated double bond in long-chain fatty acid might be crucial for the toxicity obtained. 2,4-decadienal, another major constituent in PSO, was previously reported to show significant nematicidal activity against *Meloidogyne javanica*, with an EC_50_ being 11.7 mg/L after 1 day of the treatment (Caboni et al., 2012). Similarly, the present results also identified (2*E*,4*E*)-2,4-decadienal (LC_50_ = 5.28 μg/aphid) as a potent aphicide despite weaker than both PSO (LC_50_ = 1.28 μg/aphid) and positive control thiamethoxam (LC_50_ = 1.55 μg/aphid). (9Z,12*Z*)-9,12-octadecadienoic acid occupied the second largest percentage (20.21%) in PSO, and displayed excellent and much more potent toxicity to aphids in comparison with other major compounds, which might largely underlie the aphicidal activity of PSO. However, more future work should be performed to evaluate the aphicidal activity of other minor compounds in PSO and the synergistic effects among them. Numerous previous studies have demonstrated that the overall biological effects of botanical extracts can be the result of combinations of substances with synergistic, additive, or antagonistic activity (Stermitz et al., 2000; Wagner and Ulrich-Merzenich, 2009; Junio et al., 2011).

## Conclusion

In the progress of our search for eco-friendly insecticides from natural resources, we investigated the aphicidal activity and chemical compositions of PSO for the first time. The present study revealed that PSO has significant residual and contact toxic effects against *A. craccivora*. The reduction of AChE, GST, and the total protein content in aphids treated with PSO might be responsible for the mortality of *A. craccivora*. GC-MS chemical composition analysis depicted that PSO contained long-chain fatty acid (ester), monoterpene, aldehyde, long-chain alkene, and ketone as the main categories of constituents. In addition, as the second abundant compound in PSO, (9Z,12*Z*)-9,12-octadecadienoic acid demonstrated excellent and more potent aphicidal activity than PSO, which might be a key compound toxic to aphids in PSO. However, further experimental research should be put forth to assess the aphicidal activity of other minor constituents in PSO, their synergistic effects, and the insecticidal spectrum of PSO. Meanwhile, the field trials, the effects on other enzymes like P450 and CarE, and the transcriptome-based investigation will be helpful to commercialize the PSO as a biocontrol agent against aphids.

## Data availability statement

The original contributions presented in the study are included in the article/**Supplementary Material**. Further inquiries can be directed to the corresponding author.

## Author contributions

WL: Methodology, Investigation, Writing-original draft. KK: Investigation, Writing-original draft. MH: Methodology, Data analysis. YL: Investigation, Data analysis. ZO: Methodology, Writing-review & editing. ZZ: Data analysis, Funding acquisition. YJ: Conceptualization, Funding acquisition. JZ: Project administration, Supervision, Writing-review & editing.

## Funding

This research work was financially supported by the National Natural Science Foundation of China (No. 31860091; No. 22261002), the Key Research Project of Jiangxi Province (No. 20203BBF63028), the Major Science and Technology R&D Project of Jiangxi Province (No. 20194ABC28007), the “Double Thousand Talents Plan” of Jiangxi province (jxsq2019102029), and the Science and Technology Bureau of Ganzhou City (No. 201960, No. 202060).

## Conflict of interest

The authors declare that the research was conducted in the absence of any commercial or financial relationships that could be construed as a potential conflict of interest.

## Publisher’s note

All claims expressed in this article are solely those of the authors and do not necessarily represent those of their affiliated organizations, or those of the publisher, the editors and the reviewers. Any product that may be evaluated in this article, or claim that may be made by its manufacturer, is not guaranteed or endorsed by the publisher.
