# Supplementary material for: Pomelo seed oil: Natural insecticide against cowpea aphid
Source: Front Plant Sci. 2022 Nov 8;13:1048814. doi: 10.3389/fpls.2022.1048814 (PMC9681153; doi:10.3389/fpls.2022.1048814)
Supplement: Supplementary file 1 [file DataSheet_1.docx]

**Supplementary material**

**Pomelo seed oil: Natural insecticide against cowpea aphid**

**Wei Ling^1†^, Kumaravel Kaliaperumal^1,4†^, Meiling Huang^1^, Yan Liang^1^, Zhigang Ouyang^1^, Zhonggao Zhou^3^, Yueming Jiang^2^, Jun Zhang^1,2*^**

^1^National Engineering Research Center of Navel Orange, Gannan Normal University, Ganzhou 341000, PR China

^2^South China Botanical Garden, Chinese Academy of Science, Guangzhou, 510650, PR China

^3^School of Chemistry and Chemical Engineering, Gannan Normal University, Ganzhou 341000, PR China

^4^Unit of Biomaterials Division, Department of Orthodontics, Saveetha Dental College and Hospitals, Saveetha University, Chennai, India

*** Correspondence:**Jun Zhang
[bri71527152@outlook.com](mailto:bri71527152@outlook.com)


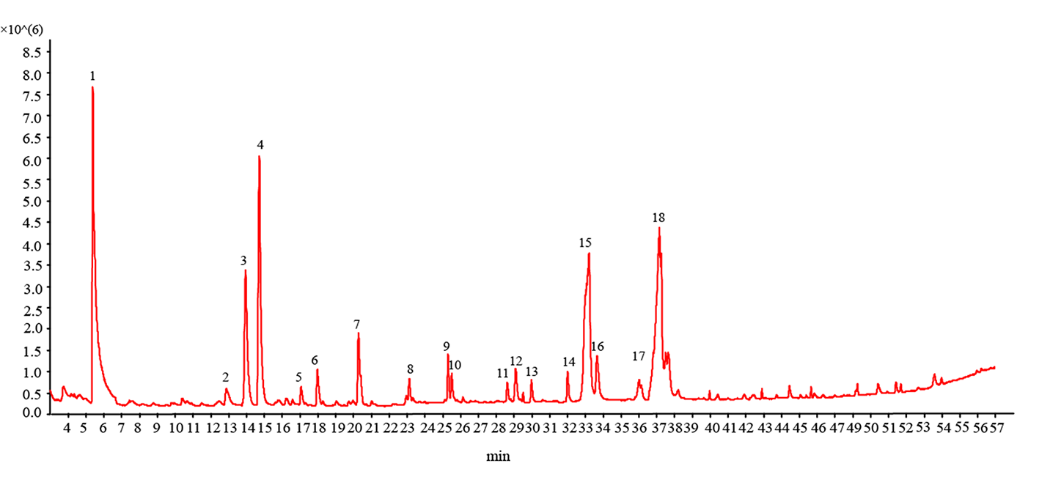


**Figure S1**. Total ion chromatogram of PSO (pomelo seed oil) (**1**, Limonene; **2**, (*E*)-2-Decenal; **3**, (2*E*,4*Z*)-2,4-Decadienal; **4**, (2*E*,4*E*)-2,4-Decadienal; **5**, Tetradecene; **6**, Caryophyllene; **7**, Eremophilene; **8**, Cetene; **9**, (6*Z*,9*E*)-Heptadecadiene; **10**, 8-Heptadecene; **11**, 1-Octadecene; **12**, Nootkatone; **13**, 6,10,14-Trimethyl-2-pentadecanone; **14**, *n*-Hexadecanoic acid methyl ester; **15**, *n*-Hexadecanoic acid; **16**, *n*-Hexadecanoic acid ethyl ester; **17**, (9*Z*,12*Z*)-9,12-Octadecadienoic acid methyl ester; **18**, (9*Z*,12*Z*)-9,12-Octadecadienoic acid).
